# Body mass index, occupational activity, and leisure-time physical activity: an exploration of risk factors and modifiers for knee osteoarthritis in the 1946 British birth cohort

**DOI:** 10.1186/1471-2474-14-219

**Published:** 2013-07-24

**Authors:** Kathryn R Martin, Diana Kuh, Tamara B Harris, Jack M Guralnik, David Coggon, Andrew K Wills

**Affiliations:** 1 Laboratory of Epidemiology and Population Sciences, National Institute on Aging, National Institutes of Health, 7201 Wisconsin Avenue, Room 3C309, Bethesda, Maryland 20814, USA; 2 MRC Unit for Lifelong Health and Ageing, University College London, London, UK; 3 Department of Epidemiology and Public Health, Division of Gerontology, University of Maryland, Maryland, USA; 4 MRC Lifecourse Epidemiology Unit, University of Southampton, Southampton, UK; 5 MRC CAiTE, School of Social & Community Medicine, University of Bristol, Bristol, UK

**Keywords:** Knee osteoarthritis, Body mass index, Physical activity, Occupational activity

## Abstract

**Background:**

Knee osteoarthritis (kOA) risk is increased by obesity and physical activities (PA) which mechanically stress the joint. We examined the associations of midlife kOA with body mass index (BMI) and activity exposure across adult life and their interaction.

**Methods:**

Data are from a UK birth cohort of 2597 participants with a clinical assessment for kOA at age 53. At ages 36, 43 and 53 BMI (kg/m^2^), self-reported leisure-time PA, and occupational activity (kneeling/squatting; lifting; climbing; sitting; assigned using a job-exposure matrix) were ascertained. Associations were explored using the multiplicative logistic model.

**Results:**

BMI was strongly and positively associated with kOA in men and women. Men and women in manual occupations also had greater odds of kOA; there was a weak suggestion that kOA risk was higher among men exposed to lifting or kneeling at work. For men, the only evidence of a multiplicative interaction between BMI and activities was for lifting (*p =* 0.01) at age 43; BMI conferred higher kOA risk among those most-likely to lift at work (OR per increase in BMI z-score: 3.55, 95% CI: 1.72-7.33). For women, the only evidence of an interaction was between BMI and leisure-time PA (*p =* 0.005) at age 43; BMI conferred higher kOA risk among those at higher PA levels (OR per increase in BMI z-score: 1.59, 95% CI: 1.26-2.00 in inactive; 1.70, 95% CI: 1.14-2.55 (less-active); and 4.44; 95% CI: 2.26-8.36 (most-active).

**Conclusions:**

At the very least, our study suggests that more active individuals (at work and in leisure) may see a greater reduction in risk of kOA from avoiding a high BMI than those less active.

## Background

High body mass index (BMI) [1-3] and physical activities involving repetitive motions and high forces such as kneeling/squatting [4-11], climbing [6-8,12], and heavy lifting [4,6-8,12] are important risk factors for knee osteoarthritis (OA). Mechanical loading and its related structural damage are thus considered the main mechanisms of knee OA (kOA) [13-16]. Given recent trends demonstrating a global increase in mean BMI over the past 30 years [17], it is important to consider whether the influence of BMI on kOA is dependent on other potentially modifiable risk factors such as occupational and leisure-time physical activity across life course.

When considering activity as a modifier of risk conferred from BMI, what matters most for public health is not the relative risk but the attributable or absolute risk from exposure to both elevated BMI and strenuous activity. Thus the combination of two independent positive associations for each factor in a multiplicative model (e.g., logistic regression, where the odd ratio for exposure to both is obtained from the product of the two odds ratios) confers a greater risk in an additive sense even if there is no evidence for a positive multiplicative interaction. A positive multiplicative interaction (in which the odds ratio for combined exposure was significantly greater than the product of odds ratios for each exposure individually) would suggest a particularly deleterious effect from exposure to both. While studies have shown that BMI and occupational activity are independently associated with kOA risk in a multiplicative model [6,7,18,19], only one has shown evidence for a positive multiplicative interaction [6]. Studies examining both physical activity and BMI are less convincing [20-25]; only one found evidence of a multiplicative interaction [26] and some report no evidence of an association between physical activity and kOA independent of BMI [23,25]. Many of these studies have been retrospective or case–control [6,7,18,20,22], potentially suffering from differential recall bias which would inflate associations. The timing of exposure may also be important. For example, a higher risk of kOA has been reported among individuals participating in vigorous activity from 20–29 years [27], and findings from our group suggest that the risk of OA accumulates from BMI through adulthood but particularly in mid-adulthood for both men and women [28]. To our knowledge there are no prospective population based studies examining the intersecting nature of BMI with activity at particular ages in midlife and few studies have examined this relationship with relatively early onset of kOA.

The objectives of this study were to examine the influence of occupational and leisure activity over adult life on risk of kOA and to test whether these activities modify the association between BMI and prevalence of relatively early onset kOA at age 53 in a UK population-based birth cohort study.

## Methods

### Sample

The Medical Research Council (MRC) National Survey of Health and Development, or 1946 British birth cohort study, is a socially stratified birth cohort of 5362 individuals who have been followed-up since their birth in 1946 with regular data collections. A total of 3035 participants (1472 men, 1563 women) participated at age 53, with the majority (n = 2989) being interviewed and examined in their own homes by trained research nurses. Contact was not attempted for the 1979 individuals who had either previously refused to take part, were living abroad, were untraced since last contact at age 43 or had already died. Participants who had a clinical examination and assessment of kOA at age 53 (n = 2597) and are the focus of this paper. The data collection received ethical approval from the MRC Ethics committee; informed consent has been given by respondents at each wave.

### Outcome

#### Knee Osteoarthritis

We established kOA status using the American College of Rheumatology criteria for a clinical diagnosis of idiopathic kOA [29]. During the home visit, research nurses queried participants on whether or not they had pain and/or stiffness in either knee on most days for at least one month in the last year, as well as physically assessed if participants had at least two of the following signs: crepitus, bony tenderness, and/or bony enlargement (please see Additional file 1) [28].

### Independent variables

#### Body mass

Height and weights were measured using standardised protocols at ages 36, 43 and 53. BMI, defined as weight (kg)/height(m)^2^, was calculated for each age, standardised by its sex specific distribution and converted to z-scores using the Lambda (variance) Mu (mean) Sigma (skewness) method [30].

#### Leisure activity levels

Activity levels were obtained at ages 36, 43 and 53. Questions asked at age 36 were based on the Minnesota leisure-time physical activity questionnaire (i.e., participation in any of 27 activities such as swimming, jogging). At ages 43 and 53, participants reported whether they had participated in any sports, vigorous leisure activities or exercises and how many months in the year and how often in these months they did each of the activities reported. For each time-point, we categorized levels of activity into three groups: inactive (no participation in relevant activities); less active (participation reported in 1–4 times in previous month/4 weeks); and most active (participation reported ≥5 times in previous month/4 weeks) [31].

#### Occupational activity exposure levels

Participants were asked about their occupation at 35, 43, and 53 years and assigned a Standard Occupational Classification (SOC) system code [32] at each time-point (see Additional file 1: Table S1). At each age, we categorized participants as having a manual or non-manual occupation using the Registrar General’s Social Classification (RGSC) of occupation. We assigned the likelihood of specific occupational exposures using a job-exposure matrix, informed by reports of occupational exposures in different job categories from two earlier studies [7,33]. The matrix consists of five occupational activities typically carried out during an average working day: kneeling or squatting for more than one hour in total; lifting weights > 25 kg by hand; walking >2 miles; climbing ladders or >30 flights of stairs; and sitting >2 hours in total. Within the job exposure matrix, occupations are categorized based on the likely frequency of exposure among workers: unlikely exposure to activity; less than 50% of workers in the occupation likely to be exposed; 50% or more of workers likely to be exposed. Because these categories refer to a probability of exposure, we refer to the three categories as ‘unlikely’, ‘somewhat likely’, and ‘highly likely’ throughout. We included in the sample those women who were classified as a ‘homemaker’ and not part of traditional, paid employment by assigning an occupational risk grade based on a review of occupation activity risk found in fifteen occupations with components found in homemaking (e.g., primary/nursery teachers, cleaners, launderers, care assistants).

### Covariables

We selected a set of potential confounders that might influence the relationship between kOA, BMI, and activity: gender, health risk factors, and individual measures of socioeconomic position. Health status at age 53 identifies participants reporting a diagnosis of one or more of the following health conditions within the previous ten years: diabetes, cancer, epilepsy or cardiovascular disease. Family history of arthritis was self-reported at ages 36 and 43, and distinguished those having either parent with arthritis at either time-point from others. History of ever having a knee injury that required medical/doctor attention was self-reported at age 53. Education was assessed as the highest level of educational attainment achieved by age 26 and grouped into five categories for all analyses: no qualifications; sub GCE or vocational course; GCE ‘O’ Level or its equivalent usually taken at age 16; GCE ‘A’ Level or its equivalent usually taken at age 18; and degree or higher. Household income at 53 years was reported by participants and three categories were used in analyses: <£24,999; £25,000-£49,999; >£50,000. Childhood social class was assessed as father’s highest attained occupational status and was categorized into three groups using the RGSC: I or II (professionals and managers); III skilled non-manual or III skilled manual; and IV or V (semi-skilled or unskilled occupations).

### Statistical analysis

Descriptive statistics were produced and distributions of all variables were explored by gender; the association between BMI and each occupational activity and leisure-time physical activity exposure was also examined. We used logistic regression to model the log odds of kOA from effect of BMI and ran separate analyses in men and women due to the documented gender differences in occupational activity exposures and risk of kOA [2,34,35]. Our first set of models, (‘minimally adjusted’) included BMI and either occupational or leisure activity at each age. In a second set of models (‘adjusted’), we adjusted for potential confounders (gender, health risk factors and socioeconomic position). We present the results from the adjusted models because the results from the minimally adjusted models were similar. We tested for a deviation from multiplicativity of odds ratios for each activity exposure with BMI (i.e., a multiplicative interaction) using a likelihood ratio test. Where there was evidence for an interaction, we present results stratified in two ways to examine: 1) the association of BMI (per z-score) with risk of kOA within stratum of activity and 2) the association of activity with risk of kOA at three nominal levels of the continuous BMI measure (−1SD, 0SD, +1SD). We used the maximum available sample for each of our models.

Within the general population, a person’s occupational exposure over adulthood is likely to be influenced by selection into and out-of occupations according to physical fitness and health, potentially causing reverse causality bias. We explored this possibility further in a sensitivity analysis by restricting the sample to those who maintained the same occupational exposure at each age (i.e., job may have changed occupational code, but assigned exposure value remained the same) and those that maintained the same leisure time activity-level at each age (36y, 43y and 53y). STATA 10.1 (StataCorp, College Station, TX) was used for all analysis.

## Results

Of the 2957 participants with a clinical knee examination, 302 (10.2%) were classified with kOA (Table 1). In general, participants’ BMI increased as they got older, from 24.1 (SD: 3.7) at 36y to 25.4 (4.2) and 27.4 (4.0) at 43y and 53y, respectively. Most self-reported good health at 53y and 22.8% reported ever having a knee injury. Nearly 36% of participants had educational qualifications of ‘A- levels’ or higher and 35% had a household income of £25,000 or more.

**Table 1:** Description of the cohort, stratified by gender

	**Total**		**Men**		**Women**		
	**Mean (SD)**	**N**	**Mean (SD)**	**N**	**Mean (SD)**	**N**	**p-value**^**a**^
Body mass index (kg/m^2^) at 36y	24.1 (3.7)	2646	24.8 (3.2)	1289	23.5 (3.9)	1357	<0.001
Body mass index (kg/m^2^) at 43y	25.4 (4.2)	2745	25.7 (3.5)	1336	25.2 (4.7)	1409	0.001
Body mass index (kg/m^2^) at 53y	27.4 (4.8)	2917	27.4 (4.0)	1438	27.4 (5.5)	1479	0.812
	**%**	**N**	**%**	**N**	**%**	**N**	**p-value**^**a**^
Knee osteoarthritis clinical observation at 53y							
Yes	10.2	302	7.5	109	12.8	193	<0.001
Disabling/life-threatening health conditions at 53y	11.9	353	11.6	169	12.2	184	0.604
1 or more							
Family history of arthritis							
Yes	39.3	1099	35.5	486	42.9	613	<0.001
History of knee injury							
Yes	22.8	673	27.5	399	18.2	274	<0.001
Education at 26y							
Degree or higher	9.9	277	14.8	203	5.2	74	
GCE ‘A’ level, Burnham B or A2	25.6	714	28.0	384	23.3	330	
GCE ‘O’ level or Burnham C	20.1	561	14.6	200	25.5	361	
Sub GCE or vocational course	7.5	208	5.9	82	8.9	126	
No qualifications	36.9	1031	36.7	504	37.2	527	<0.001
Income at 53 y							
>£50,000	8.4	234	9.7	137	7.0	97	
£25,000-£49,999	26.1	732	27.7	391	24.5	341	
<£24,999	65.5	1835	62.6	882	68.5	953	0.002

^a^*p-*values for gender comparison by means of t-test & chi square.

Around a third of participants were employed in a ‘manual’ occupation at each time-point (Table 2). Lifting and kneeling occupational activities were more common than climbing or walking, and approximately 70% of participants were in occupations which involved sitting for ≥ two hours/day. The prevalence of occupational activity exposures remained relatively stable with age, except for kneeling which decreased with age. The majority of participants had low-levels of leisure-time physical activity and the prevalence of inactivity increased with age. Compared with women, men had a higher mean BMI, more reports of knee injuries, and were more likely to engage in lifting and kneeling at work at ages 36 and 43. In comparison, women had a higher prevalence of kOA, disabling/life-threatening health conditions, family history of arthritis, and were more likely to be inactive at ages 36 and 43 and to be in occupations involving sitting.

**Table 2:** Cohort activity exposure (occupational /leisure) at 36, 43, and 53 years, stratified by gender

	**36 years**		**43 years**		**53 years**	
	**Men**	**Women**	**Men**	**Women**	**Men**	**Women**
	**% (N)**	**% (N)**	**% (N)**	**% (N)**	**% (N)**	**% (N)**
Manual occupation	68.3 (586)	31.7 (272)	36.1 (457)	24.4 (284)	39.1 (564)	28.9 (430)
Non-manual occupation	57.7 (702)	42.3 (513)	63.9 (810)	75.6 (880)	60.9 (877)	71.1 (1058)
*p-value*^*a*^		<0.001		<0.001		<0.001
Lifting^§^ unlikely	58.9 (773)	76.9 (1001)	61.3 (793)	71.1 (975)	62.6 (858)	71.8 (965)
Lifting somewhat likely	21.3 (279)	14.1 (183)	21.8 (282)	18.6 (255)	21.2 (291)	16.1 (217)
Lifting highly likely	19.8 (260)	9.0 (117)	16.9 (218)	10.4 (142)	16.1 (221)	12.1 (163)
*p-value*^*a*^		<0.001		<0.001		<0.001
Kneeling^§^ unlikely	61.9 (812)	45.5 (592)	63.3 (819)	60.6 (831)	67.7 (927)	66.3 (891)
Kneeling somewhat likely	22.4 (294)	46.6 (606)	21.7 (280)	31.1 (427)	18.4 (252)	24.5 (330)
Kneeling highly likely	15.7 (206)	7.9 (103)	15.0 (194)	8.3 (114)	13.9 (191)	9.2 (124)
*p-value*^*a*^		<0.001		<0.001		<0.001
Walking^§^ unlikely	90.8 (1191)	98.2 (1278)	93.0 (1203)	98.2 (1347)	92.1 (1262)	98.1 (1319)
Walking likely	9.2 (9.22)	1.8 (23)	7.0 (90)	1.8 (25)	7.9 (108)	1.9 (26)
*p-value*^*a*^		<0.001		<0.001		<0.001
Climbing^§^ unlikely	90.0 (1181)	96.0 (1249)	90.9 (1175)	95.6 (1312)	91.1 (1248)	94.7 (1273)
Climbing somewhat likely	6.3 (82)	4.0 (52)	5.5 (71)	4.4 (60)	5.0 (69)	5.3 (72)
Climbing highly likely	3.7 (49)	0 (0)	3.6 (47)	0 (0)	3.9 (53)	0 (0)
*p-value*^*a*^		<0.001		<0.001		<0.001
Sitting^§^ unlikely	31.2 (409)	22.4 (291)	31.2 (403)	25.5 (350)	30.2 (414)	28.8 (287)
Sitting somewhat likely	26.8 (351)	51.9 (675)	28.0 (363)	36.0 (494)	24.2 (332)	27.0 (375)
Sitting highly likely	42.0 (552)	25.7 (335)	40.8 (527)	38.5 (528)	45.6 (624)	43.4 (583)
*p-value*^*a*^		<0.001		<0.001		0.096
Physical activity						
Inactive	31.2 (405)	41.3 (565)	47.5 (642)	55.0 (784)	48.1 (699)	50.7 (762)
Less active	26.5 (344)	24.5 (335)	23.5 (318)	23.0 (328)	18.6 (270)	16.1 (242)
Most active	42.4 (551)	34.3 (469)	29.0 (392)	12.0 (313)	33.3 (484)	33.2 (499)
*p-value*^*a*^		<0.001		<0.001		0.163

^a^*p-*values for gender comparison by means of chi square or Fisher’s exact test.

^§^ Lifting: Regular lifting of weights ≥ 25 kg by hand; Kneeling: Bending, kneeling or squatting; Sitting: Sitting >2 hours per day; Climbing: Climbing ladders or >30 flights of stairs; Walking: Walking >2 miles per day.

An examination of the unadjusted distributions of BMI by activity (Additional file 1: Table S2) indicated that individuals in non-manual occupations consistently had higher BMI while those in manual occupations had a lower BMI at ages 36, 43 and 53. Associations between BMI and occupational activities were observed for various activities (e.g., lifting, kneeling, sitting and exercise), but these were generally small and inconsistent. There was a wide range of BMI scores within each stratum of physical activity, indicating there was a necessary amount variation in BMI needed to further examine whether physical activity modifies the association between BMI and kOA.

### Associations in men

In models containing both BMI and each activity domain: BMI was generally positively associated with kOA, such that for every z-score increase in BMI, the odds of kOA increased by an approximate factor of 1.4 (OR range 1.40-1.47), and men in manual occupations had a 2-fold increase in odds of kOA when compared to those in non-manual occupations (95% CI: 1.19, 3.49). There was a weak suggestion that men who were exposed to lifting or kneeling at work at age 53 had a higher risk of kOA. There was no evidence for an association with any of the other occupational exposures, or leisure activity at any ages (Table 3).

**Table 3:** Adjusted associations of BMI and activity (occupational/ leisure) at 36, 43, and 53 years with kOA in men

	**36 years**		**43 years**		**53 years**	
	**OR**	***p*-value**	**OR**	***p*-value**	**OR**	***p*-value**
Manual occupation	0.98 (0.55, 1.76)	0.955	1.27 (0.74, 2.17)	0.386	2.03 (1.19, 3.49)	0.010
Non-manual occupation	Referent		Referent		Referent	
BMI (per z-score)	1.22 (0.95, 1.56)	0.117	1.47 (1.14, 1.89)	0.003	1.44 (1.15, 1.82)	0.002
p-value for test of interaction		0.350		0.232		0.758
Lifting unlikely	1.15 (0.62, 2.17)	0.654	0.71 (0.41, 1.24)	0.226	0.57 (0.33-0.99)	0.044
Lifting somewhat likely	Referent		Referent		Referent	
Lifting highly likely	1.18 (0.57, 2.47)	0.659	0.62 (0.30, 1.27)	0.190	0.83 (0.43-1.60)	0.572
BMI (per z-score)	1.20 (0.93, 1.54)	0.153	1.41 (1.09, 1.81)	0.008	1.40 (1.10-1.77)	0.006
p-value for test of interaction		0.133		0.011		0.745
Kneeling unlikely	1.17 (0.63, 2.15)	0.621	0.96 (0.54, 1.70)	0.887	0.60 (0.34-1.05)	0.072
Kneeling somewhat likely	Referent		Referent		Referent	
Kneeling highly likely	0.93 (0.42, 2.07)	0.868	0.58 (0.26, 1.29)	0.180	0.76 (0.37-1.54)	0.446
BMI (per z-score)	1.19 (0.93, 1.53)	0.163	1.41 (1.09, 1.82)	0.008	1.39 (1.10-1.76)	0.006
p-value for test of interaction		0.557		0.348		0.807
Sitting unlikely	0.92 (0.47, 1.77)	0.792	0.88 (0.49, 1.58)	0.670	1.00 (0.56-1.78)	0.999
Sitting somewhat likely	Referent		Referent		Referent	
Sitting highly likely	1.13 (0.61, 2.06)	0.700	0.69 (0.39, 1.24)	0.226	0.60 (0.34-1.07)	0.085
BMI (per z-score)	1.19 (0.93, 1.53)	0.164	1.41 (1.09, 1.81)	0.008	1.42 (1.12-1.78)	0.004
p-value for test of interaction		0.171		0.278		0.874
Inactive	1.47 (0.76, 2.86)	0.255	1.07 (0.62, 1.85)	0.800	1.77 (0.90-3.48)	0.095
Less active	Referent		Referent		Referent	
Most active	1.59 (0.85, 2.96)	0.145	0.78 (0.42, 1.47)	0.447	1.44 (0.71-2.88)	0.309
BMI (per z-score)	1.31 (1.03, 1.66)	0.027	1.46 (1.16, 1.84)	0.001	1.42 (1.14-1.77)	0.002
p-value for test of interaction		0.307		0.284		0.476

*OR* Odds Ratio, *BMI* Body Mass Index.

**Figure 1:**
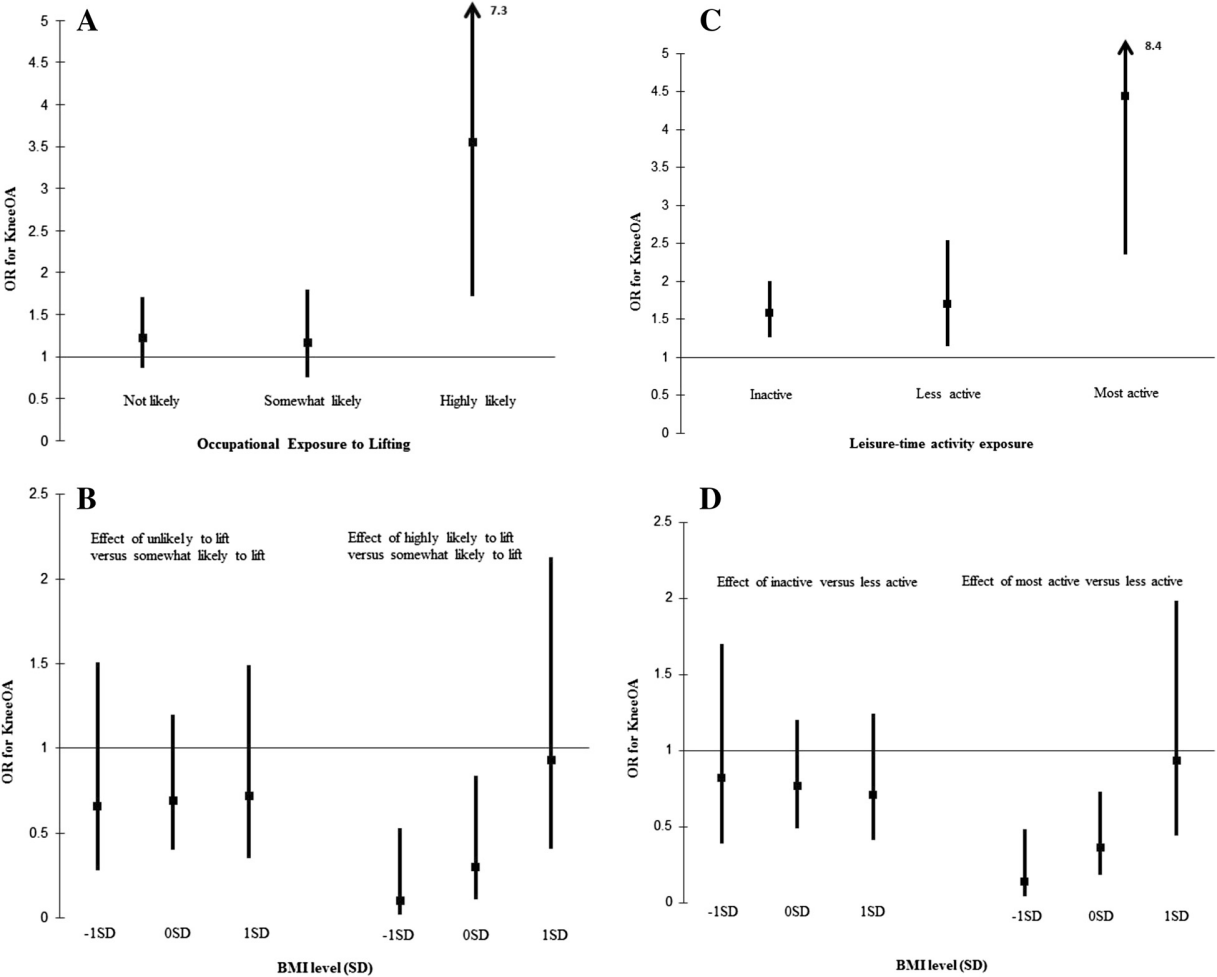
**Results for interaction between BMI and activity exposure. A** - Occupational Exposure to Lifting among men; **B** – BMI level (SD) among men; **C** – Leisure-time activity exposure among women; **D** – BMI level (SD) among women.

Note: separate models were examined for each activity exposure and BMI and coefficients reported are from models without a BMIxActivity interaction, adjusting for potential confounders - education at 26y, income at 53y, childhood social class, family history of arthritis, history of knee injury and health status at 53y. Where there was evidence for an interaction, Figures 1A and 1B show the full stratified odds ratios for BMI and activity in these models.

We found no evidence of a multiplicative interaction between BMI and occupational status, occupational exposure to kneeling/bending and sitting, or leisure activity influencing the risk of kOA among men at ages 36, 43, or 53 (Table 3). The only evidence of an interaction between activity and BMI in men occurred between occupational lifting and BMI (*p =* 0.011) at 43y (Table 3). The effect of BMI (per z-score) on kOA within stratum of lifting indicated that BMI conferred greater odds of kOA for men employed in occupations that were ‘highly likely’ to involve lifting (OR: 3.55, 95% CI: 1.72-7.33), while there was no effect of BMI on risk of kOA observed among men ‘unlikely’ or ‘somewhat likely’ to lift (Figure 1A). Comparing occupations ‘highly likely’ to lift versus those ‘somewhat likely’ to lift, we interpreted the interaction in terms of the effect of lifting on risk of kOA at the three levels of BMI (−1SD, 0SD, and +1SD). Results suggested that an inverse association of lower odds for kOA comparing occupations ‘highly likely’ to lift versus those ‘somewhat likely’ to lift, was only present among those with lower BMI (−1SD - OR**:** 0.14, 95% CI: 0.03-0.60; 0SD - OR: 0.30, 95% CI: 0.11-0.84; +1SD – OR: 0.93, 95% CI: 0.41-2.13) (Figure 1B).

### Associations in women

In models containing both BMI and each activity domain: BMI was generally positively associated with kOA (OR ~ 1.8; OR range 1.49-1.92), there was a suggestion of a reduced risk of kOA for women ‘highly likely’ to sit compared to those in occupations ‘somewhat likely’ to sit 2 or more hours per day at age 36 (OR: 0.56, 95% CI: 0.33-0.94) and at age 43 (OR: 0.57,95% CI: 0.36-0.89), and women in manual occupations at ages 36 and 53 had an approximate 85% increase in odds of kOA when compared to those in non-manual occupations. There was no evidence for an association between any of the other domains of activity and kOA in women.

There was no evidence of a multiplicative interaction between BMI and any of the occupational exposures among women (Table 4). However, there was evidence of an interaction between BMI and leisure activity (*p =* 0.005) at age 43 (Table 4), such that BMI conferred greater odds of kOA as levels of reported activity increased. For every increase per z-score of BMI, the odds of kOA increased by an approximate factor of 1.59 (95% CI: 1.26-2.00) for ‘inactive’ women, 1.70 (95% CI: 1.14-2.55) for ‘less active’ women, and 4.44 (95% CI: 2.26-8.36) for ‘most active’ women (Figure 1C). Comparing activity levels ‘most active’ versus those ‘less active’, we interpreted the interaction in terms of the effect of leisure activity on risk of kOA at the three levels of BMI (−1SD, 0SD, and +1SD). Results suggested that ‘most active’ women had the most benefit in terms of reduced risk of kOA when compared to those ‘less active’ women if they had lower levels of BMI (−1SD - OR: 0.14, 95% CI: 0.04-0.48 and 0SD - OR: 0.36, 95% CI: 0.18-0.73; +1SD – OR: 0.93, 95% CI: 0.44-1.98) (Figure 1D).

**Table 4:** Adjusted associations of BMI and activity (occupational/ leisure) at 36, 43, and 53 years with kOA in women

	**36 years**		**43 years**		**53 years**	
	**OR**	**p-value**	**OR**	**p-value**	**OR**	**p-value**
Manual occupation	1.85 (1.06, 3.24)	0.031	1.09 (0.66, 1.82)	0.734	1.87 (1.22, 2.86)	0.004
Non-manual occupation	Referent		Referent		Referent	
BMI (per z-score)	1.49 (1.16, 1.91)	0.002	1.73 (1.39, 2.15)	<0.001	1.92 (1.59, 2.33)	<0.001
p-value for test of interaction		0.691		0.570		0.203
Lifting unlikely	0.89 (0.51, 1.55)	0.681	1.10 (0.67, 1.81)	0.710	0.71 (0.42, 1.19)	0.317
Lifting somewhat likely	Referent		Referent		Referent	
Lifting highly likely	0.73 (0.32, 1.66)	0.449	1.09 (0.55, 2.18)	0.070	1.02 (0.51, 2.07)	0.590
BMI (per z-score)	1.80 (1.47, 2.21)	<0.001	1.85 (1.52, 2.25)	<0.001	1.73 (1.49, 2.24)	<0.001
p-value for test of interaction		0.344		0.580		0.808
Kneeling unlikely	0.74 (0.49, 1.11)	0.143	0.79 (0.52, 1.18)	0.248	0.93 (0.58, 1.51)	0.776
Kneeling somewhat likely	Referent		Referent		Referent	
Kneeling highly likely	0.91 (0.45, 1.85)	0.793	0.80 (0.41, 1.59)	0.530	1.23 (0.58, 2.61)	0.582
BMI (per z-score)	1.78 (1.45, 2.18)	<0.001	1.83 (1.50, 2.22)	<0.001	1.91 (1.29, 2.83)	<0.001
p-value for test of interaction		0.295		0.780		0.405
Sitting unlikely	1.15 (0.72, 1.84)	0.549	0.76 (0.47, 1.24)	0.276	1.29 (0.77, 2.16)	0.343
Sitting somewhat likely	Referent		Referent		Referent	
Sitting highly likely	0.56 (0.33, 0.94)	0.029	0.57 (0.36, 0.89)	0.013	0.89 (0.56, 1.43)	0.653
BMI (per z-score)	1.77 (1.45, 2.18)	<0.001	1.80 (1.48, 2.19)	<0.001	1.48 (1.41, 2.22)	<0.001
p-value for test of interaction		0.292		0.846		0.234
Inactive	1.00 (0.63, 1.60)	0.985	0.73 (0.47, 1.13)	0.153	1.46 (0.87, 2.47)	0.154
Less active	Referent		Referent		Referent	
Most active	0.99 (0.61, 1.63)	0.978	0.50 (0.28, 0.91)	0.022	0.67 (0.37, 1.21)	0.184
BMI (per z-score)	1.85 (1.51, 2.25)	<0.001	1.82 (1.50, 2.20)	<0.001	1.84 (1.52, 2.23)	<0.001
p-value for test of interaction		0.118		0.005		0.414

*OR* Odds Ratio, *BMI* Body Mass Index.

Note: separate models were examined for each activity exposure and BMI and coefficients reported are from models without a BMIxActivity interaction, adjusting for potential confounders - education at 26y, income at 53y, childhood social class, family history of arthritis, history of knee injury and health status at 53y. Where there was evidence for an interaction, Figures 1C and 1D show the full stratified odds ratios for BMI and activity in these models.

### Absolute risk

Table 5 shows the estimated additive risk of kOA from combinations of exposure to high BMI and manual occupation with kOA. It illustrates that joint exposure to both may carry an extra additive risk despite the absence of an interaction in the logistic regression model.

**Table 5:** **Absolute risk difference for kOA from exposure to a higher BMI, manual occupation and combination at 53y**^**†**^

		**Men**		**Women**	
		**Non-manual**	**Manual**	**Non-manual**	**Manual**
		% (95% CI)	% (95% CI)	% (95% CI)	% (95% CI)
BMI (z-score)	0	Reference	+ 6.6 (2.0,14.6)	Reference	+ 8.8 (6.1,12.7)
	+1	+3.0 (1.0,5.3)	+ 11.8 (4.1,22.7)	+9.2 (6.8,16.8)	+ 21.7 (12.2,32.8)

^†^Absolute risk difference represented as % (95% CI); based on baseline risk of 7.5% and 12.8% in men and women respectively (see Table 1). Adjusted model based estimates (logistic); adjusted for potential confounders - education at 26y, income at 53y, childhood social class, family history of arthritis, history of knee injury and health status at 53y.

### Sensitivity analysis on selection

Only 237 men and 144 women had the same occupational activity exposure at ages 36, 43 and 53 and the number individuals with kOA was very small (men: n = 15; women: n = 15). This resulted in reduced analytic power to detect any associations with occupational activity or leisure activity. Qualitatively, however, the results in terms of main effects of BMI and activity exposures were similar.

## Discussion

Our findings from a population-based prospective cohort study suggest that men and women in manual occupations are at an increased risk of kOA, and that men exposed to lifting or kneeling at work in later adulthood may have a higher risk of kOA. Higher BMI was consistently associated with an elevated risk of kOA. Lastly, there was some evidence of a multiplicative interaction between BMI and lifting in men such that the positive association between BMI and kOA was strongest in those likely to be exposed to regular lifting of 25 kg at age 43, and in women, where BMI conferred a higher odds ratio for kOA among the most-active women, and conversely there was a protective association of higher activity among women with lower levels of BMI.

Our study has several strengths, namely we were able to examine associations gathered at three stages in adulthood using prospective data, which should minimise recall bias. In addition, symptomatic cases of kOA were determined via a standardised clinical examination. We also examined this relationship in a younger population which may be less prone to comorbidities that would bias our findings. And unlike other studies [18], we included ‘homemakers’ (women not in paid employment) in our analyses. Finally, while our results may suffer from bias due to loss to follow-up (e.g., loss of contact, emigration, survey-wave refusal, permanent refusal, death), it is difficult to conceive of a mechanism that would radically alter the associations between exposures and outcome considered here among those lost to follow up. Further, the sample at age 53 remained largely representative of a similarly-aged UK-born population [36,37], and as such the nature and level of the exposures studied should be generalizable to the wider UK population of similar age.

A few limitations should also be noted. While a standardized clinical examination protocol was used, it is possible that observer error was introduced into the examination, possibly leading to systematic miss-classification of kOA cases. In addition, our cases are prevalent rather than incident since they were obtained by a screening only at age 53. As such, we cannot exclude the possibility that the exposures post-dated the onset of disease, although this is unlikely for exposures at ages 36 and 43 given the reported age-related incidence of kOA rises steeply after age 50 [38,39]. Unknown is whether individuals exited their occupation and entered into less physically-demanding work due to physically limiting health conditions. Our attempt to examine this was limited due to the small-sample size of individuals with the same occupational exposure across time. And while there were benefits to drawing upon an existing job-exposure matrix, developed specifically to examine knee-risk exposure from occupational activity, the accuracy of a job-exposure matrix is limited by the specificity of the occupational categories upon which it is based. If the occupational categories are broad, as is normally the case in general population-based studies, there will be heterogeneity of exposure within occupational categories, and not all individuals within a group will be accurately classified. Compared to methods that ascertain individual-level exposure, some additional measurement error is likely from this group-based approach. Therefore, possible errors in case ascertainment and assignment of exposure may have obscured the associations with risk factors in this study, especially given the previous support for occupational activities as a risk factor for kOA [6,7,18,19]. Our results may not be generalizable to current or future populations given the changing BMI landscape and occupational activity levels.

The greater observed odds of kOA among those in manual occupations may be due to the aggregate exposure to a range of higher risk activities undertaken by those in manual occupations. This is supported in our study by the suggestive associations between lifting and kneeling and kOA in men and the protective association between sitting at work and kOA in women, which agrees with previous research showing a link between physically arduous activities at work and kOA [6,18,26]. While our finding that sitting at work may offer protection from kOA in women has been shown before [8], it seems unlikely that sitting is the causal factor that reduces the risk of kOA. Rather this more likely reflects the fact that individuals who sit more at work are less-often exposed to strenuous occupational activity that increases the risk of kOA through higher mechanical loads [19,40,41].

Our finding of a multiplicative interaction between lifting and BMI in men is supported by a previous case–control study [6], although not by another prospective study [19]. However, it is important to note that out of 30 interaction tests that we performed, only two were statistically significant at the 5% level and not all tests can be considered as independent. It is thus possible that these two reflect chance rather than any true underlying multiplicative interaction between exposure to activity and BMI. McWilliams et al. [42] noted in a recent meta-analysis examining occupational risk factors for kOA that there was evidence of publication bias, such that cross-sectional and case–control studies more often report greater risk of kOA from occupational activity than do prospective or longitudinal studies. However both interactions were evident at age 43 and if we ignore the age 53 results as being most likely to be biased towards the null due to healthy worker and reverse causality bias (i.e., individuals self-selecting out of physically demanding occupations and subsequently gaining weight), then this would suggest that an individual has less sensitivity, in terms of kOA risk, to exposure to higher levels of BMI and activity in younger adulthood (interactions were also not observed at age 26 – results not shown).

The evidence for a combination of independent multiplicative effects for BMI and activity seen in our study and in particular the absence of any negative interactions emphasizes the potential public health importance of joint exposure to high BMI and high activity stressors. Any evidence for a positive interaction would add to the public health importance of these exposures. Our findings with regard to these possible synergistic effects on kOA require further investigation. Large-scale prospective studies are required to further investigate these relationships. Individual-level, direct measures of occupational activities should be used to characterize the population with greater resolution and examine the extreme ends of the activity spectrum. For example, one study suggests a non-linear pattern - only those with the very heaviest exposure to physical activity at work are at risk of kOA [19]. Future prospective studies would also do well to incorporate clinical and radiographic assessment of kOA at multiple time-points past age 40 to better determine incident and prevalent cases.

## Conclusion

In conclusion, while we found some evidence that risk of kOA conferred from BMI was more pronounced among more active individuals, our results were only suggestive. Further investigation using prospective study designs and individual-level activity exposure ascertainment is warranted, especially given the importance of these risk factors alongside the growing obesity epidemic and declining activity levels. At the very least, our study suggests that more active individuals (at work and in leisure) may see greater benefit (i.e. in lower risk of kOA) from avoiding a high BMI than those who are less active. Developing a better understanding of the relationship between BMI, physical activity and kOA has definite public health implications for how activity is safely prescribed so that populations at greater risk can be appropriately targeted for tailored community-level or work-place health interventions.

## Abbreviations

BMI: Body mass index; kOA: Knee osteoarthritis; UK: United Kingdom; SOC: Standard occupational classification; RGSC: Registrar general’s social classification; GCE: General certificate of education; SD: Standard deviation; OR: Odds ration; CI: Confidence interval.

## Competing interests

The authors declare that they have no competing interests.

## Authors' contributions

KRM and AKW conducted analyses and drafted the manuscript. All authors have contributed to the conception and design, have been involved in revising the manuscript critically for important intellectual content, and all have given final approval of this version to be published.

## Pre-publication history

The pre-publication history for this paper can be accessed here:

http://www.biomedcentral.com/1471-2474/14/219/prepub

## Supplementary Material

Additional file 1 **A. Clinical knee examination protocol. B.** ACR Criteria for clinical classification of idiopathic osteoarthritis (OA) of the knee [29]. C. **Table S1.** Top 20 Occupations for men and women by year, ranked from high to low. d. **Table S2.** Distribution of BMI by activity exposure (occupational/leisure) at age 36y, 43y and 53y, by gender. Click here for file
