# Supplementary material for: Body mass index, occupational activity, and leisure-time physical activity: an exploration of risk factors and modifiers for knee osteoarthritis in the 1946 British birth cohort
Source: BMC Musculoskelet Disord. 2013 Jul 24;14:219. doi: 10.1186/1471-2474-14-219 (PMC3726290; doi:10.1186/1471-2474-14-219)
Supplement: Additional file 1 — A. Clinical knee examination protocol. B. ACR Criteria for clinical classification of idiopathic osteoarthritis (OA) of the knee [29]. C. Table S1. Top 20 Occupations for men and women by year, ranked from high to low. d. Table S2. Distribution of BMI by activity exposure (occupational/leisure) at age 36y, 43y and 53y, by gender. [file 1471-2474-14-219-S1.docx]

**Additional file 1**

**Clinical knee examination protocol:**

The clinical knee examination was conducted with the participant in a seated position, with their legs stretched out in front resting on another chair. Protocol dictated that the examination could be conducted over leggings, tights or most trousers, although trousers rolled up above the knee, so long as to not restrict movement, was preferred. Nurses were instructed to examine the knee for evidence of crepitus, bony tenderness, bony enlargement and evidence of varus or valgus knee alignment in the following ways.

Crepitus (passive) was assessed with the nurse placing a left hand over the knee cap and the right hand holding the ankle at the front, flexing the knee from full extension to a 90^0^ flexion. The left hand was to feel for a grating sensation during the movement and again as the knee is straightened to full extension. Any palpated grating was designated as crepitus.

Bony tenderness was assessed via palpation of the antero-medial and antero-lateral joint margins with the left and right thumbs as the knee was flexed at 90^0^. Bony tenderness is considered present when, at uniform pressure, tenderness of the joint margin at the level of the patella but medial or lateral to it is noted by participant expression of discomfort.

Bony enlargement was assessed via palpation around the circumference of the knee joint margin for evidence of craggy cold hard swelling representing osteophyte from the femoral condyles or tibule plateau.

**ACR Criteria for clinical classification of idiopathic osteoarthritis (OA) of the knee [30]:**

Knee pain +

at least 3 of 6:

Age>50 years

Stiffness <30 minutes

Crepitus

Bony tenderness

Bony enlargement

No palpable warmth

**Table S1: Top 20 Occupations for men and women by year, ranked from high to low**

| Men | |  |  |  |  |
| --- | --- | --- | --- | --- | --- |
|  | 1982 |  | 1989 |  | 1999 |
|  | SOC^*^ Code and Occupation Title |  | SOC Code and Occupation Title |  | SOC Code and Occupation Title |
|  |  |  |  |  |  |
| 1. | 234 Primary and nursery education teach |  | 179 Managers and proprietors in service |  | 179 Managers and proprietors in service |
| 2. | 110 Production, works and maintenance |  | 139 Other financial institutions and of |  | 872 Drivers of road goods vehicles |
| 3. | 179 Managers and proprietors in service |  | 199 Other managers and administrators |  | 121 Marketing and sales managers |
| 4. | 430 Clerks (n.o.s.) |  | 872 Drivers of road goods vehicles |  | 516 Metal working production and maintenance |
| 5. | 872 Drivers of road goods vehicles |  | 234 Primary and nursery education teach |  | 233 Secondary education teachers |
| 6. | 139 Other financial institutions |  | 430 Clerks (n.o.s.) |  | 110 Production, works and maintenance |
| 7. | 516 Metal working production and maintenance |  | 516 Metal working production and maintenance |  | 504 Builders, building contractors |
| 8. | 521 Electricians, electrical maintenance |  | 121 Marketing and sales managers |  | 521 Electricians, electrical maintenance |
| 9. | 160 Farm owners and managers, horticulture |  | 110 Production, works and maintenance m |  | 570 Carpenters and joiners |
| 10. | 121 Marketing and sales managers |  | 521 Electricians, electrical maintenance |  | 139 Other financial institutions and of |
| 11. | 710 Technical and wholesale sales rep. |  | 504 Builders, building contractors |  | 874 Taxi, cab drivers and chauffeurs |
| 12. | 320 Computer analyst/programmers |  | 710 Technical and wholesale sales rep. |  | 111 Managers in building contracting |
| 13. | 504 Builders, building contractors |  | 570 Carpenters and joiners |  | 199 Other managers and administrators n |
| 14. | 600 NCOs and other ranks, UK armed forces |  | 160 Farm owners and managers, horticulture |  | 720 Sales assistants |
| 15. | 610 Police officers (sergeant and below) |  | 210 Civil, structural, municipal |  | 210 Civil, structural, municipal |
| 16. | 210 Civil, structural, municipal |  | 610 Police officers (sergeant and below) |  | 532 Plumbers, heating and ventilating e |
| 17. | 570 Carpenters and joiners |  | 540 Motor mechanics, auto engineers |  | 160 Farm owners and managers, horticulture |
| 18. | 441 Storekeepers, warehousemen/women |  | 111 Managers in building contracting |  | 594 Gardeners, grounds men/women |
| 19. | 532 Plumbers, heating and ventilating |  | 120 Treasurers and company financial manager |  | 507 Painters and decorators |
| 20. | 240 Judges and officers of the court |  | 175 Publicans, innkeepers and club steward |  | 873 Bus and coach drivers |
|  |  |  |  |  |  |
|  |  |  |  |  |  |
| Women | |  |  |  |  |
|  | 1982 |  | 1989 |  | 1999 |
|  | SOC^*^ Code and Occupation Title |  | SOC Code and Occupation Title |  | SOC Code and Occupation Title |
|  |  |  |  |  |  |
| 1. | 1111 Homemaker |  | 430 Clerks (n.o.s.) |  | 459 Other secretaries, personal assistants |
| 2. | 430 Clerks (n.o.s.) |  | 234 Primary and nursery education teach |  | 430 Clerks (n.o.s.) |
| 3. | 452 Typists and word processor operator |  | 452 Typists and word processor operator |  | 720 Sales assistants |
| 4. | 234 Primary and nursery education teach |  | 1111 Homemaker |  | 234 Primary and nursery education teach |
| 5. | 720 Sales assistants |  | 720 Sales assistants |  | 958 Cleaners |
| 6. | 340 Nurses |  | 340 Nurses |  | 644 Care assistants and attendants |
| 7. | 659 Other childcare and related occupations |  | 958 Cleaners |  | 410 Accounts and wages clerks, book-keepers |
| 8. | 958 Cleaners |  | 659 Other childcare and related occupations |  | 233 Secondary education teachers |
| 9. | 179 Managers and proprietors in service |  | 179 Managers and proprietors in service |  | 340 Nurses |
| 10. | 460 Receptionists, general office |  | 293 Social workers, probation officers |  | 1111 Homemaker |
| 11. | 622 Bar staff |  | 953 Catering assistants |  | 179 Managers and proprietors in service |
| 12. | 660 Hairdressers, barbers coiffeur |  | 490 Computer operators, data processing |  | 460 Receptionists, general office |
| 13. | 953 Catering assistants |  | 641 Hospital ward assistants |  | 420 Filing, computer and other records |
| 14. | 231 Higher and Further education teachers |  | 199 Other managers and administrators n |  | 371 Welfare, community and youth worker |
| 15. | 641 Hospital ward assistants |  | 721 Retail cash desk and check-out operator |  | 659 Other childcare and related occupation |
| 16. | 651 Playgroup leaders |  | 231 Higher and Further education teacher |  | 411 Counter clerks and cashiers |
| 17. | 952 Kitchen porters, hands |  | 460 Receptionists, general office |  | 620 Chefs, cooks hotel supervisor |
| 18. | 620 Chefs, cooks hotel supervisor |  | 139 Other financial institutions and of |  | 652 Educational assistants |
| 19. | 621 Waiters, waitresses |  | 660 Hairdressers, barbers coiffeur |  | 139 Other financial institutions and of |
| 20. | 174 Restaurant and catering managers |  | 862 Packers, bottlers, canners, fillers |  | 953 Catering assistants |
|  |  |  |  |  |  |

* SOC code is 1990 SOC code

**Table S2: Distribution of BMI by activity exposure (occupational/leisure) at age 36y, 43y and 53y, by gender.**

|  | 36 years | |  | 43 years | |  | 53 years | |
| --- | --- | --- | --- | --- | --- | --- | --- | --- |
|  | Men | Women |  | Men | Women |  | Men | Women |
|  | Mean (95% CI) | Mean (95% CI) |  | Mean (95% CI) | Mean (95% CI) |  | Mean (95% CI) | Mean (95% CI) |
|  |  |  |  |  |  |  |  |  |
| Manual occupation | 24.38 (24.19, 24.58) | 22.94 (22.70, 23.18) |  | 25.35 (25.15, 25.56) | 24.27 (24.04, 24.51) |  | 27.07 (26.82, 27.32) | 27.3 (26.30, 26.87) |
| Non-manual occupation | 24.90 (24.64, 25.16) | 23.46 (23.05, 23.88) |  | 25.73 (25.43, 26.03) | 25.53 (25.04, 26.03) |  | 27.24 (26.90, 27.60) | 27.7 (27.28, 28.33) |
| *p-value** | *p=*0.002 | *p=*0.014 |  | *p=*0.035 | *p*≤0.001 |  | *p=*0.405 | *p*≤0.001 |
|  |  |  |  |  |  |  |  |  |
| Lifting^§^ unlikely | 24.33 (24.12, 24.53) | 23.27 (23.05, 23.49) |  | 25.30 (25.09, 25.51) | 24.54 (24.30, 24.78) |  | 27.03 (26.78, 27.29) | 26.85 (26.55, 27.15) |
| Lifting somewhat likely | 25.08 (24.74, 25.42) | 23.26 (22.81, 23.72) |  | 25.59 (25.21, 25.96) | 25.07 (24.54, 25.62) |  | 27.15 (26.72, 27.59) | 27.25 (26.59, 27.92) |
| Lifting highly likely | 24.85 (24.85, 24.49) | 23.29 (22.68, 23.91) |  | 25.96 (25.53, 26.40) | 25.40 (24.69, 26.12) |  | 27.73 (27.17, 28.30) | 26.81 (26.10, 27.54) |
| *p-value* | *p*≤0.001 | *p=*0.999 |  | *p=*0.016 | *p=*0.018 |  | *p=*0.067 | *p=*0.527 |
|  |  |  |  |  |  |  |  |  |
| Kneeling^§^ unlikely | 24.48 (24.28, 24.68) | 23.01 (22.75, 23.27) |  | 25.35 (25.14, 25.55) | 24.48 (24.23, 24.73) |  | 27.12 (26.87, 27.37) | 26.61 (26.30, 26.93) |
| Kneeling somewhat likely | 24.72 (24.37, 25.07) | 23.43 (23.14, 23.72) |  | 25.75 (25.36, 26.15) | 24.78 (24.38, 25.18) |  | 27.68 (27.18, 28.19) | 27.17 (26.68, 27.66) |
| Kneeling highly likely | 24.87 (24.47, 25.26) | 23.74 (23.03, 24.47) |  | 25.61 (25.16, 26.08) | 26.33 (25.50, 27.18) |  | 26.77 (26.24, 27.31) | 28.26 (27.28. 29.27) |
| *p-value* | *p=*0.164 | *p=*0.036 |  | *p=*0.136 | *p*≤0.001 |  | *p=*0.041 | *p*≤0.001 |
|  |  |  |  |  |  |  |  |  |
| Walking^§^ unlikely | 24.59 (24.42, 24.75) | 23.26 (23.07, 23.44) |  | 25.49 (25.31, 25.66) | 24.71 (24.50, 24.92) |  | 27.18 (26.96, 27.39) | 26.95 (26.69, 27.21) |
| Walking likely | 24.66 (24.15, 25.17) | 24.03 (22.83, 25.30) |  | 25.31 (24.71, 25.93) | 25.41 (23.73, 27.20) |  | 27.07 (26.29, 27.87) | 25.03 (23.68, 26.45) |
| *p-value* | *p=*0.798 | *p=*0.281 |  | *p=*0.598 | *p=*0.378 |  | *p=*0.781 | *p=*0.040 |
|  |  |  |  |  |  |  |  |  |
| Climbing^§^ unlikely | 24.55 (24.38, 24.71) | 23.21 (23.03, 23.40) |  | 25.46 (25.28, 25.64) | 24.64 (24.43, 24.86) |  | 27.20 (26.98, 27.42) | 26.83 (26.57, 27.09) |
| Climbing somewhat likely | 24.77 (24.08, 25.48) | 24.53 (23.46, 25.66) |  | 25.50 (24.75, 26.27) | 26.53 (25.48, 27.62) |  | 26.63 (25.62, 27.67) | 28.38 (27.01, 29.83) |
| Climbing highly likely | 25.34 (24.67, 26.04) | n/a |  | 25.77 (25.02, 26.53) | n/a |  | 27.22 (26.44, 28.01) | n/a |
| *p-value* | *p=*0.140 | *p=*0.006 |  | *p=*0.790 | *p*≤0.001 |  | *p=*0.482 | *p=*0.009 |
|  |  |  |  |  |  |  |  |  |
| Sitting^§^ unlikely | 24.82 (24.52, 25.11) | 23.52 (23.10, 23.95) |  | 25.58 (25.27, 25.89) | 25.54 (25.09, 26.00) |  | 26.83 (26.45, 27.21) | 27.39 (26.87, 27.91) |
| Sitting somewhat likely | 24.79 (24.46, 25.12) | 23.31 (23.05, 23.58) |  | 25.65 (25.32, 25.98) | 24.59 (24.24, 24.95) |  | 27.47 (27.05, 27.88) | 26.64 (26.20, 27.08) |
| Sitting highly likely | 24.31 (24.08, 24.53) | 22.96 (22.63, 23.29) |  | 25.27 (25.03, 25.52) | 24.32 (24.01, 24.61) |  | 27.24 (26.94, 27.55) | 26.79 (26.40, 27.19) |
| *p-value* | *p=*0.010 | *p=*0.109 |  | *p=*0.154 | *p*≤0.001 |  | *p=*0.070 | *p=*0.068 |
|  |  |  |  |  |  |  |  |  |
| Physical Activity |  |  |  |  |  |  |  |  |
| Inactive | 24.75 (24.45, 25.06) | 23.97 (23.65, 24.30) |  | 25.61 (25.35, 25.87) | 25.38 (25.06, 25.70) |  | 27.26 (26.95, 27.58) | 27.89 (27.49, 28.30) |
| Less Active | 24.47 (24.16, 24.78) | 22.94 (22.63, 23.24) |  | 25.72 (25.40, 26.04) | 24.23 (23.87, 24.59) |  | 27.06 (26.66, 27.46) | 26.39 (25.85, 26.94) |
| Most Active | 24.66 (24.45, 24.87) | 22.62 (22.38, 22.86) |  | 25.08 (24.80, 25.37) | 23.89 (23.55, 24.24) |  | 26.95 (26.62, 27.29) | 25.88 (25.53, 26.24) |
| *p-value* | 0.383 | <0.001 |  | 0.010 | <0.001 |  | 0.392 | <0.001 |

Note: BMI was log-transformed ; mean and CI were back-transformed

* *p-value for a test of general association*

^§^ Lifting: Regular lifting of weights ≥ 25 kg by hand; Kneeling: Bending, kneeling or squatting; Sitting: Sitting >2 hours per day; Climbing: Climbing ladders or >30 flights of stairs; Walking: Walking >2 miles per day.
